# Recombinant oncolytic adenovirus armed with CCL5, IL-12, and IFN-γ promotes CAR-T infiltration and proliferation in vivo to eradicate local and distal tumors

**DOI:** 10.1038/s41420-023-01626-4

**Published:** 2023-09-02

**Authors:** Lin Fang, Sen Yuan, Meng Wang, Chen Zhang, Xueyan Wang, Hailong Li, Jie Yang, Wanjing Li, Nan Sun, Qi Zhang, Yuxin Zhang, Dafei Chai, Huizhong Li, Junnian Zheng, Gang Wang

**Affiliations:** 1 Cancer Institute, Xuzhou Medical University, 209 Tongshan Road, Xuzhou, Jiangsu 221004 China; 2 Center of Clinical Oncology, The Affiliated Hospital of Xuzhou Medical University, 99 West Huaihai Road, Xuzhou, Jiangsu 221002 China; 3 Jiangsu Center for the Collaboration and Innovation of Cancer Biotherapy, Xuzhou Medical University, 209 Tongshan Road, Xuzhou, Jiangsu 221004 China; 4 Cancer Center of Xuzhou No.1 People’s Hospital, Xuzhou, Jiangsu China; 5 Department of Oncology, The First People’s Hospital of Yancheng, Xuzhou, Jiangsu China

**Keywords:** Cancer immunotherapy, Tumour immunology

## Abstract

The efficacy of chimeric antigen receptor T (CAR-T) cells for solid tumors remains unsatisfactory due to the limited tumor infiltration and immunosuppressive microenvironment. To overcome these limitations, the genetically engineered recombinant oncolytic adenoviruses (OAVs) that conditionally replicate in tumor cells were developed to modify the tumor microenvironment (TME) to facilitate CAR-T-mediated tumor eradication. Here in the present study, a novel recombinant OAV carrying CCL5, IL12, and IFN-γ controlled by Ki67 promoter was constructed (named AdKi67-C3). The antitumor activity of AdKi67-C3 was tested in vitro and in vivo by using mono administration or combing with CAR-T cells targeting B7H3. It proved that CCL5 expressed by AdKi67-C3 indeed induced more CAR-T migration in vitro and CAR-T infiltration in tumor mass in vivo. Meanwhile, cytokines of IFN-γ and IL12 secreted by AdKi67-C3-infected tumor cells significantly promoted proliferation and persistence of CAR-T cells in vitro and in vivo. In tumor-bearing xenograft mouse models of kidney, prostate or pancreatic cancer, local pretreatment with AdKi67-C3 dramatically enhanced CAR-T cell efficacy and eliminated local and distant tumors. More importantly, mice achieving complete tumor regression resisted to re-challenge with the same tumor cells, suggesting establishment of long-term antitumor immune response. Therefore, OAVs armored with cytokines could be developed as a bioenhancer to defeat the immunosuppressive microenvironment and improve therapeutic efficacy of CAR-T in solid tumors.

## Introduction

Chimeric antigen receptor T cell (CAR-T) is one of the significant achievements of tumor immunotherapy [1]. Although it has achieved great success in hematological cancers [2–4] and autoimmune diseases [5–7], CAR-T therapy has met huge challenges in treating solid tumors [8]. There are several factors restricting its therapeutic efficacy of CAR-T in solid tumors, such as insufficient infiltration cell number, limited persistent time, immunosuppressive tumor microenvironment, ect [9–11]. To overcome those limitations, multiple strategies have been developed, modifying CAR-T cells with matrix-degrading enzymes [12] or chemokine receptors [13] or self-secreting cytokines [14] to enhance tumor infiltration and prolong survival time, taking use of auxiliary therapy to reverse the immunosuppressive tumor microenvironment [15–17], and so on.

Oncolytic viruses (OVs) are a kind of virus which can selectively replicate in tumor cells and destroy them [18]. Increasing studies have shown that OVs could be used as an ectopic gene delivering vector to enhance the antitumor immune responses in TME [19, 20]. Talimogene laherparepvec (T-VEC) is the first US FDA approved OV expressing granulocyte-macrophage colony-stimulating factor [21] which increased CD8^+^ T density on recurrence-free survival of patients with advanced resectable melanoma [22]. TILT-123 is an OV carrying tumor necrosis factor alpha and interleukin-2 that rewired the ovarian tumor microenvironment to accommodate heightened antitumor TIL reactivity [23]. These findings indicate that OVs could be used as an adjuvant in combination with other therapies, such as immune checkpoint inhibitors and CAR-T cells [24–26].

In solid tumors, insufficient infiltration of tumor-specific T cells might be attributed to unfavorable chemokine gradients [15]. Reduction of chemokine (C-C motif) ligand 5 (CCL5) resulted in reduced tumor-infiltrating lymphocyte (TIL) cells. In turn, overexpression of CCL5 or C-X-C motif chemokine ligand 9 (CXCL9) was positively associated with CD8^+^ T cell infiltration in solid tumors [27]. The proliferation and the long-term survival of tumor-infiltrating T cells in tumor mass also contributes to the antitumor activity of adoptive immune cell therapy. Interleukin-12 (IL-12) produced by activated macrophages, monocytes, dendritic cells, or B lymphocytes [28] could induce T-helper type1 (Th1) immunity and cytolysis [29]. Furthermore, IL-12 stimulates production of interferon (IFN)-γ from T and natural killer (NK) cells [30, 31]. IL-12 can elicit potent antitumor activity by inducing an immunomodulatory effect.

In order to improve the therapeutic efficacy of CAR-T cells in solid tumors, an oncolytic adenovirus-based bioenhancer armed with chemokine CCL5, cytokines IL-12, and IFN-γ was designed and constructed, named Ki67-C3, in which a tumor-specific promoter Ki67 was used to drive the adenoviral E1A expression to control virus replication. Intratumoral administration of Ki67-C3 displayed complete regression of tumor in tumor-bearing xenograft mice while combining with B7H3-specific CAR-T cells. Importantly, locally pretreatment with Ki67-C3 facilitated B7H3-CAR-T to eradicate distant tumors and prevent tumor recurrence. Therefore, our study provides theoretical basis for the clinical application of OV-based bioenhancer to improve CAR-T efficacy in solid tumors.

## Results

### CD3^+^ tumor-infiltrating lymphocytes (TILs) expressed increased chemokine receptors uniquely in human renal cancer

**Fig. 1:**
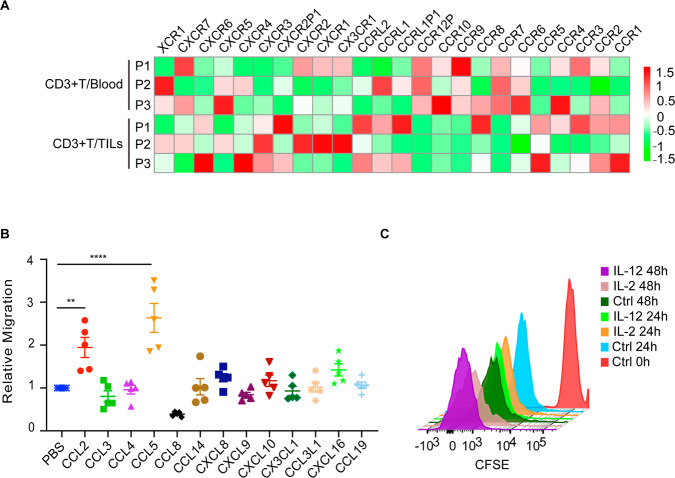
Increased chemokine receptors existed on CD3+ tumor-infiltrating lymphocytes (TILs) in human renal cancer. **A** Expression profile of chemokine receptors in CD3^+^T cells infiltrated in tumors and CD3^+^T cells in blood generated from human renal cancers and matched blood specimens (n = 3). Red indicates high expression, and green indicates low expression. **B** The migration of CAR-T cells was evaluated by adding different kinds of T-cell chemotactic factor using a transwell migration assay. **C** B7H3-CAR-T cells were stained with CFSE (10 mM) and were co-cultured with IL2 (500 U/mL), IL12 (20 ng/mL) individually or X-vivo medium only. The cells were analyzed by flow cytometry on day 0 (CFSE-labeling), day 1 and day 2. Data represent mean ± SEM. ** means *P* < 0.01, ***P < 0.001.

In order to explore the difference of T cells in peripheral blood and in tumor tissues, three human renal cancer tissues and paired blood specimens were collected. Second-generation sequencing was performed with purified CD3-positive T cells. RNA sequencing data showed that chemokine receptors expression such as CCR5 and CXCR3 was significantly higher in CD3^+^T cells infiltrated in tumors compared with those in blood (Fig. 1A). Given the key role of chemokine receptors in facilitating T cell infiltration, we identified the chemotaxis of several chemokines. The data showed that among all chemokines, chemokine CCL5 promoted more activated T cells to migrate into the lower chamber (Fig. 1B). It had been reported that tumor cells downregulate CCL5 expression through DNA methylation and chemokine circuitries to restrict T-cell infiltrating in tumors [27]. It is necessary to rescue the loss of CCL5 expression in human tumors. We also verified that IL12 could promote CAR-T cells proliferation by CFSE staining assay, even more potent than that of IL2 (Fig. 1C).

### Oncolytic adenovirus efficiently infected tumor cells and expressed interested genes

**Fig. 2:**
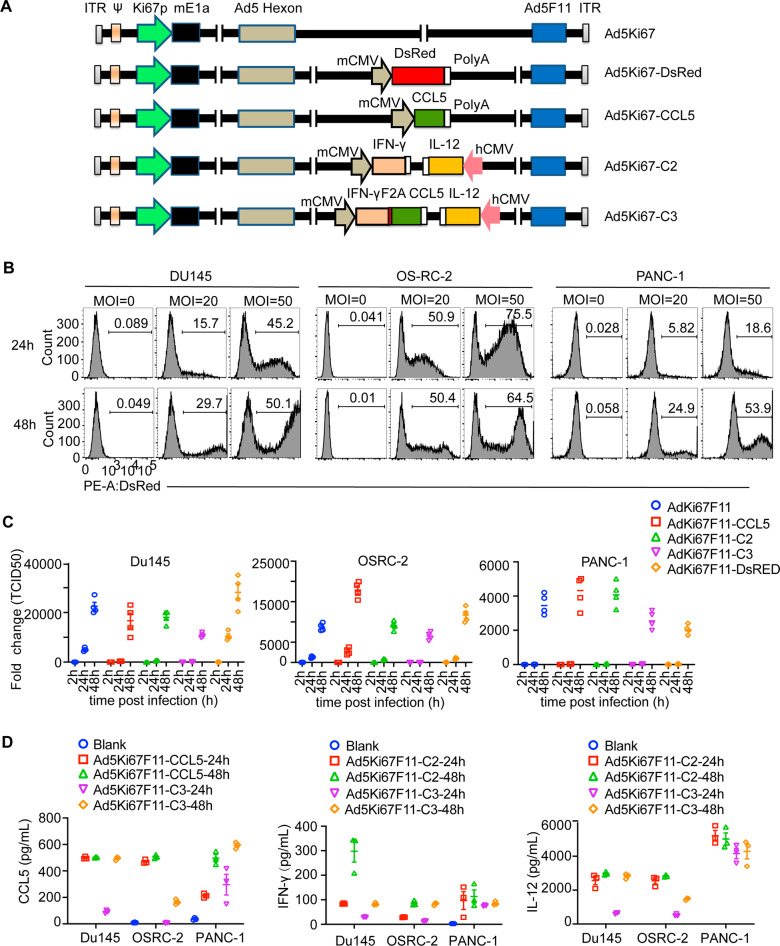
Generation of series of Ad5Ki67 recombinant adenovirus, and infection, replication and genes expression of Ad5Ki67 in tumor cells. **A** Scheme showing the construction of Ad5Ki67 recombinant adenoviruses expressing DsRed, CCL5, IFN-γ + IL12 (C2) or CCL5 + IFN-γ + IL12 (C3). **B** OAV Ad5Ki67-DsRed can replicate and multiply normally in tumor cells. Du145, OSRC-2 and PANC-1 cells were infected with Ad5Ki67-DsRed at a MOI of 20 or 50 for 24 or 48 h. The cells were harvested and DsRed expression was detected by flow cytometry. **C** Du145, OSRC-2 and PANC-1 cells were infected with different OAVs at MOIs of 20, respectively. Cells were harvested at various time points as indicated. TCID_50_ assay were preformed to detect the viral particles in cells. Folds of change were obtained by comparing the value of 2 h. **D** The supernatant was harvested from infected tumor cells. CCL5, IFN-γ or IL12 was assayed by Elisa.

To overcome the limitation of CAR-T cell infiltration, accumulation, and persistence, an oncolytic adenovirus simultaneously expressing CLL5, IFN-γ, and IL-12 (AdKi67-C3) was constructed based on the backbone of serotype 5 parental OAV, AdKi67, aiming to improve the therapeutic efficacy of CAR-T cells in solid tumors. AdKi67 was introduced by the Ki67 promoter to drive the expression of adenoviral E1A, which had been identified to possess specific oncolytic effect on tumor cells in our previous research [32]. Other OAVs as control were also constructed. AdKi67-CCL5 expressed chemokine CCL5, and AdKi67-C2 expressed cytokines IL-12 and IFN-γ. AdKi67-DsRed expressed reported gene DsRed (Fig. 2A). AdKi67-DsRed was used to determine the infection efficiency of the oncolytic adenoviruses in tumor cells. Du145, OSRC-2, and PANC-1 cells were infected with AdKi67-DsRed at the indicated MOIs. The Flow cytometry assay showed that DsRed expression was time and dose dependent. AdKi67-DsRed could efficiently infect tumor cells (Fig. 2B).

Next, the replication of the OAVs in tumor cells was detected. AdKi67, AdKi67-CCL5, AdKi67-C2, AdKi67-C3, and AdKi67-DsRed infected Du145, OSRC-2, and PANC-1 cells with 20 MOIs for 2 h, 24 h, and 48 h, respectively. The cell lysates were collected through 3 cycles of freezing and thawing. TCID50 (tissue culture infective dose 50%) assay was used to determine the progeny virus in infected cells. AdKi67, AdKi67-CCL5, AdKi67-C2, AdKi67-C3, and AdKi67-DsRed all had potent replication ability and were time-dependent in infected tumors (Fig. 2C). It was not surprising for AdKi67-C3, incorporation of transgenes modestly decreased the efficiency of virus replication in vitro. CCL5, IFN-γ, or IL-12 expression in tumor cells infected with indicated oncolytic adenovirus was also assayed by Elisa. The tumor cells infected with oncolytic adenovirus secreted large amounts of cytokines and the expression level was in a time-dependent manner (Fig. 2D). These data suggested that the genes carrying by oncolytic adenoviruses AdKi67 could efficiently expressed and secreted in several tumor cells.

### Combined AdKi67-C3 and B7H3-CAR-T cells efficiently lysed target tumor cells

**Fig. 3:**
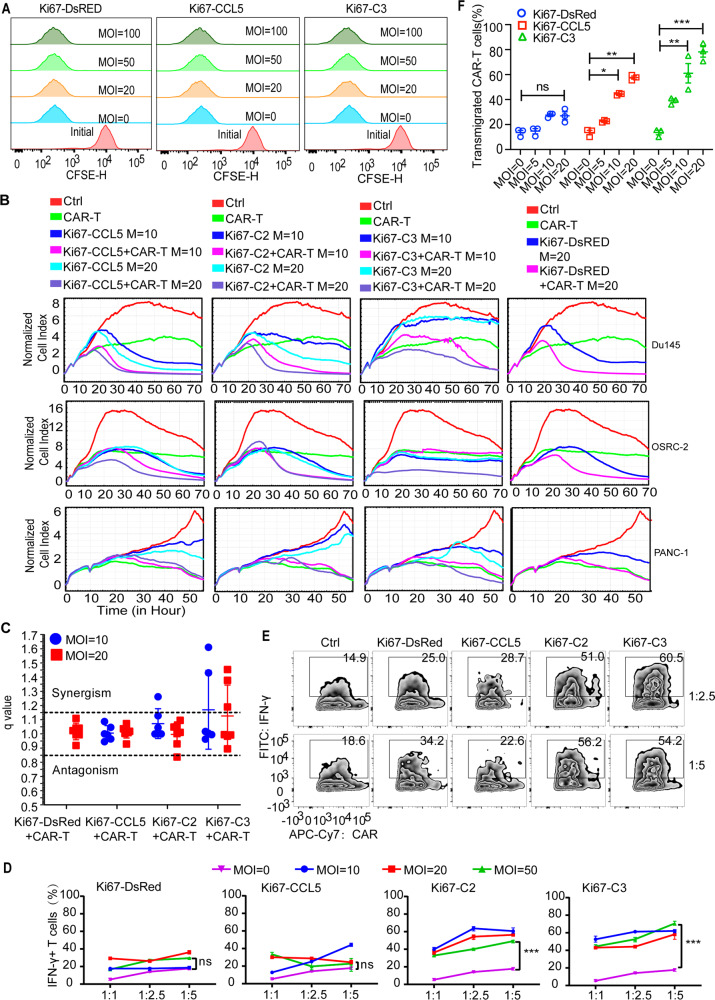
Oncolytic adenovirus Ad5Ki67-CCL5 + IFN-γ + IL12 (Ad5Ki67-C3) enhanced activation, proliferation, and lytic activity of B7H3-redirected chimeric antigen receptor T cells (B7H3-CAR-T cells). **A** B7H3-CAR T cells were stained with CFSE (10 mM) and were infected with Ad5Ki67-DsRed, Ad5Ki67-CCL5 or Ad5Ki67-C3 at indicated MOIs. B7H3-CAR-T cells were analyzed by flow cytometry. **B** Kinetics of Du145, OSRC-2 and PANC-1 tumor cell lysis incubated with the combination of Ad5Ki67-DsRed, Ad5Ki67-CCL5, Ad5Ki67-C2 or Ad5Ki67-C3 with B7H3-CAR-T cells measured by the real-time xCELLigence cell analyzer. **C** Q value plots show synergistic effects on cell viability in Ad5Ki67-C3 with B7H3-CAR-T combination treatment. Q values were calculated using the Chou–Talalay theorem. Q > 1.15, synergism; 0.85 ≤ Q ≤ 1.15, additivity; Q < 0.85, antagonism. The combination q value was calculated with a modified Burgi’s formula. **D** Du145 cells were co-cultured with different MOIs Ad5Ki67-DsRed, Ad5Ki67-CCL5, Ad5Ki67-C2 or Ad5Ki67-C3 combined with B7H3-CAR-T cells at different E: T ratio (1:1, 1:2.5 and 1:5). The IFN-γ-positive B7H3-CAR-T cells were analyzed by flow cytometry in Co-cultured B7H3-CAR-T cells. Data were from three independent experiments. **E** Representative flow cytometric analysis showing abundance of intracellular IFN-γ in B7H3-CAR-T cells after co-culture with Du145 tumor cells in the presence or absence of the indicated MOI of OAVs. **F** The migration of B7H3-CAR-T was evaluated by transwell assay. B7H3-CAR-T cells were co-cultured with the supernatant collected from control or infected Du145 cells. Data represent mean ± SEM. *P < 0.05; **P < 0.01, ***P < 0.001, ns means not significant.

We wanted to test whether AdKi67-C3 enhanced the function of B7H3-CAR-T cells in vitro. B7H3-CAR-T cells were prepared by infecting activated T cells with B7H3-CAR retroviruses. First, we checked the effect of OAVs on B7H3-CAR-T cells proliferation. B7H3-CAR-T cells were pre-stained with CFSE and co-cultured with AdKi67-DsRed, AdKi67-CCL5 or AdKi67-C3 at different MOIs. Results showed that the addition of OAVs did not significantly affect the proliferation of CAR-T cells (Fig. 3A). Subsequently, we tested whether AdKi67-C3 enhanced the lytic activity of B7H3-CAR-T cells using real-time cell analysis in vitro. Du145, OSRC-2, and PANC-1 cells were infected with AdKi67-DsRed, AdKi67-CCL5, AdKi67-C2, or AdKi67-C3 at 10 or 20 MOIs in combination with or without B7H3-CAR-T cells at effector: target (E: T) ratio of 1:2. RTCA data showed that the combination of OAVs and CAR-T cells induced efficiently more target tumor cell death than CAR-T or OAV mono group (Fig. 3B). Calculation of the Q value of endpoint from each group indicated a synergistic antitumor effect of combination therapy in AdKi67-C2 or AdKi67-C3 with B7H3-CAR-T cells (Fig. 3C).

The IFN-γ positive B7H3-CAR-T cells were also analyzed in the co-cultured context. Briefly, Du145 cells were infected with AdKi67-DsRed, AdKi67-CCL5, AdKi67-C2, or AdKi67-C3 at the indicated MOI, meanwhile, B7H3-CAR-T cells were also added at E: T ratio of 1:1, 1:2.5 or 1:5, respectively. Forty-eight hours later, the proportion of IFN-γ positive CAR-T cells was significantly increased in AdKi67-C2 or AdKi67-C3 treated group compared with the B7H3-CAR-T cells treatment group. And no increased IFN-γ-positive CAR-T cells in AdKi67-DsRed or AdKi67-CCL5 group were observed (Fig. 3D, E). Indicating that AdKi67-C2 or AdKi67-C3 pre-infection significantly improved CAR-T cell activation when cultured with tumor cells. The supernatant of Du145 cells infected with AdKi67-CCL5 or AdKi67-C3 improved the migration of B7H3-CAR-T cells determined by transwell migration assay (Fig. 3F). Suggesting that secreted CCL5 by OAV-infecting tumor cells were functional. These data showed that the combination of AdKi67-C3 and B7H3-CAR-T cells has a synergistic antitumor effect on human cancer cells in vitro.

### Combination of AdKi67-C3 with B7H3-CAR-T cells caused tumor regression in a Du145 tumor xenograft NCG mouse model

**Fig. 4:**
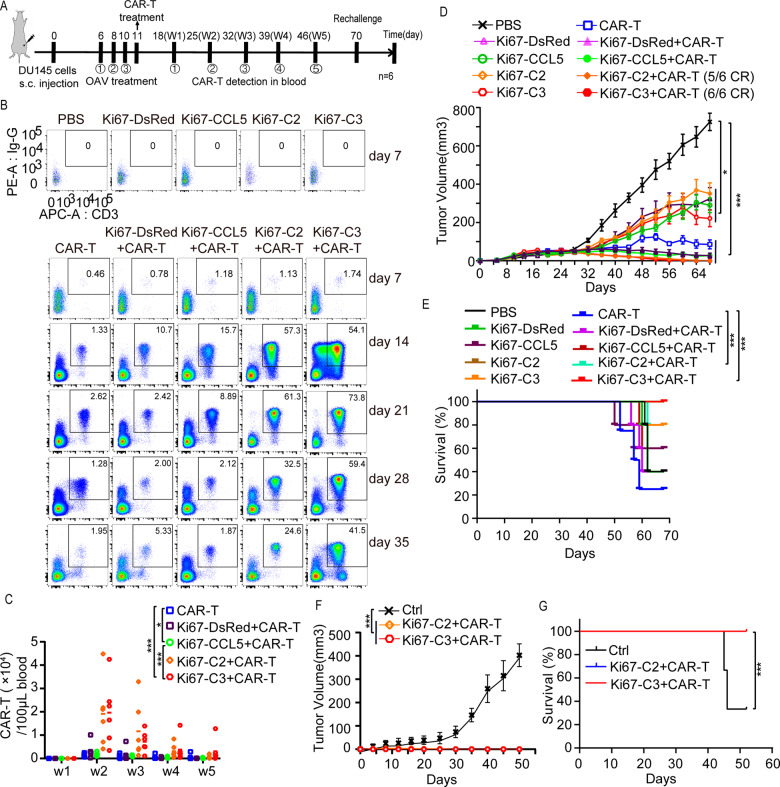
Oncolytic adenovirus Ad5Ki67-C3 enhanced antitumor efficacy of B7H3-CAR-T cells and improved survival in the Du145 xenograft model. **A** Experimental schematic. Du145 tumor-bearing mice were treated with either intratumoral injection of PBS or 1×10^9^ plaque-forming units (pfu) of virus OAVs followed by intravenous injection of either PBS or 2×10^6^ B7H3-CAR T cells at the next day after last OAVs injection. Tumor volumes were monitored by caliper measurement. **B** Quantification of B7H3-CAR positive T cells in peripheral blood from 6 mice of different treatment groups was obtained by bleeding on day 7, 14, 21 28 and 35 post B7H3-CAR-T cells infusion. The representative data were shown. **C** B7H3-CAR-T cell counts in peripheral blood were calculated by using counting beads. The data was performed the statistic assay at 2-week time point (w2). Means and SEM of plotted data at all time points were presented. **D** Tumor volumes were measured by caliper measurement. Means and SEM are shown (n = 6 each). CR, complete tumor regression. **E** Kaplan-Meier survival curve. Data are from the experiment shown in (**D**). **F** Mice that achieved complete regression (CR) against Du145 for about 55 days and age-matched treatment-naive mice were subcutaneously inoculated with Du145. Tumor growth for mice is shown (n = 6 for PBS and Ad5Ki67-C3 + CAR-T group, n = 5 for Ad5Ki67-C2 + CAR-T group). **G** Kaplan-Meier survival curve of rechallenge mice. Data are from the experiment shown in (**F**). *P < 0.05; **P < 0.01, ***P < 0.001, ns means not significant.

To evaluate the antitumor efficacy of combination therapy in vivo, we first tested AdKi67-C3 combined with B7H3-CAR-T cell therapy in a Du145 xenograft NCG mouse model, the intervention regimen is depicted in Fig. 4A. AdKi67-DsRed, AdKi67-CCL5, AdKi67-C2 or AdKi67-C3 was administrated by intra-tumor injection and B7H3-CAR-T cells were infused through tail-vein on the next day of last OAV administration on combination therapy groups. In order to monitor the survival and proliferation of B7H3-CAR-T cells in mice, blood samples were collected each group from week 1 to 5 after B7H3-CAR-T infusion. The results showed that the proportion of CAR-positive T cells in AdKi67-CCL5 + CAR-T group was higher than B7H3-CAR-T or AdKi67-DsRed+CAR-T group. Moreover, the proportion of CAR positive T cells in B7H3-CAR-T, AdKi67-DsRed+CAR-T or AdKi67-CCL5 + CAR-T group was the highest on day 14 and decreased sharply at the later stage, while AdKi67-C2 + CAR-T and AdKi67-C3 + CAR-T groups still had bigger proportion of CAR-T cells at the later stage. In B7H3-CAR-T mono, AdKi67-DsRed+CAR-T or AdKi67-CCL5 + CAR-T group, few CAR-T cells were presented in mice compared to the other combination groups. AdKi67-C3 + CAR-T group had the highest proportion of CAR-T cells in mice. As expected, there were no positive B7H3-CAR-T cells in PBS, AdKi67-DsRed, AdKi67-CCL5, AdKi67-C2 or AdKi67-C3 group (Fig. 4B, C).

We also found that B7H3-CAR-T cell monotherapy suppressed tumor growth moderately and AdKi67-DsRed, AdKi67-CCL5, AdKi67-C2 or AdKi67-C3 monotherapy weakly inhibited tumor growth. While the combination of AdKi67-C2 or AdKi67-C3 and B7H3-CAR-T cells significantly prevented tumor growth compared to PBS or monotherapy. Six of six mice treated with AdKi67-C3 + CAR-T achieved complete tumor regression (CR) and the CR ratio for AdKi67-C2 + CAR-T group was 5/6. AdKi67-DsRed+CAR-T and AdKi67-CCL5 + CAR-T also partially suppressed tumor growth compared to mono therapy, but there was no CR in these treatment groups (Fig4. D). Likewise, the combination of AdKi67-C2 or AdKi67-C3 and B7H3-CAR-T cells had improved survival of mice compared with other groups (Fig.4E).

Additionally, to examine the long-term persistence of antitumor effect, mice cured from AdKi67-C2 + CAR-T and AdKi67-C3 + CAR-T groups were rechallenged with the same DU145 cells. The age-matched naive mice inoculated with DU145 were set as control. Tumors in the cured mice were completely rejected within 55 days, whereas all age-matched tumor-naive control mice developed tumors (Fig. 4F). It was also observed that mice previously cured of tumors by AdKi67-C2 + CAR-T or AdKi67-C3 + CAR-T treatment also had longer survival compared to the control group (Fig. 4G). These data suggested that AdKi67-C2 or AdKi67-C3 significantly increased the proliferation and persistence of CAR-T cells in vivo and the combination prolonged the survival of mice. The long-term antitumor memory in AdKi67-C2 or AdKi67-C3 combined with B7H3-CAR-T cells group was also established in Du145 tumor-engrafted mice.

### The efficacy of local injection of AdKi67-C3 combined with intravenous B7H3-CAR-T in the treatment of distal tumors in vivo

**Fig. 5:**
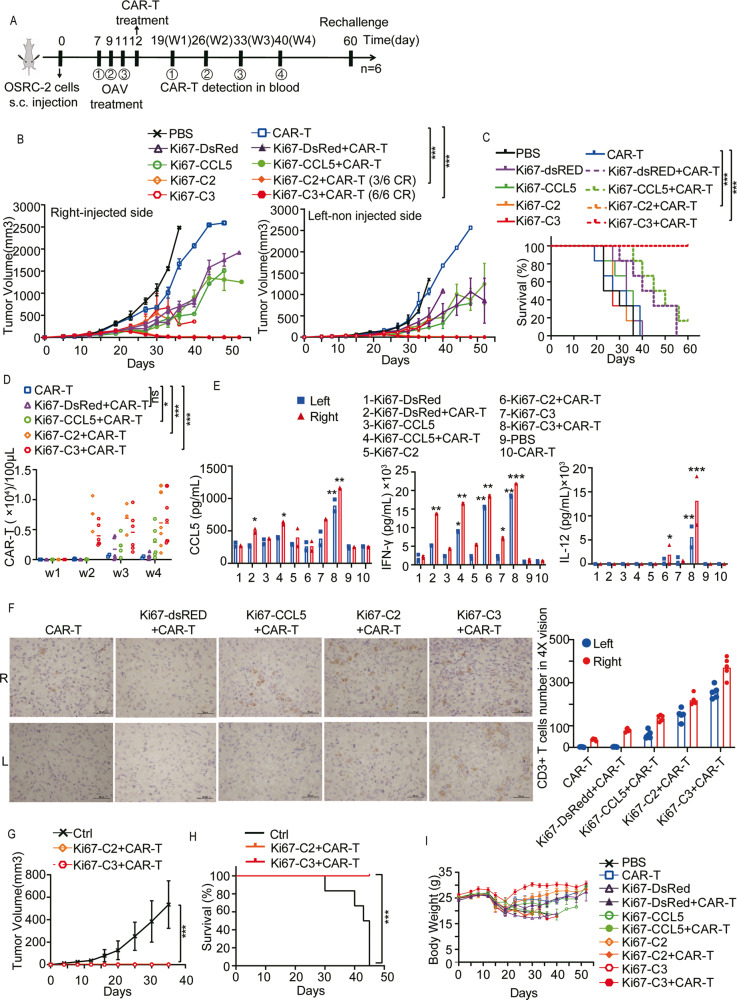
Oncolytic adenovirus Ad5Ki67-C3 combined with B7H3-CAR-T cells had abscopal antitumor effect. **A**, **B** Mice were subcutaneously inoculated with OSRC-2 cells in both flanks. After establishment of tumors, PBS, 1×10^9^ pfu of different OAVs were injected into the tumors in the right flanks (Right) every other day, for a total of three times. The combination treatment was performed by infusing B7H3-CAR-T cells on the next day of last OAV administration (**A**). Tumor volumes of both flanks were measured by caliper measurement. Means and SEM are shown (n = 6 each) (**B**). CR was defined as the complete tumor regression of bilateral tumors. **C** Kaplan-Meier survival curve. Data are from the experiment shown in (**B**). **D** 7, 14, 21 28 days after CAR-T infusion treatment, CD3^+^B7H3^+^ T cells were analyzed by flow cytometry. **E** Detection of CCL5, IFN-γ and IL-12 by Elisa in tumor homogenates collected from mice 5 days after inoculations of CAR-T. Data represent mean ± SEM in 8 mice for each virus. **F** Analysis of CD3^+^ T cells infiltrating into the bilateral tumors were assessed by immunohistochemistry (IHC) on day 5 post CAR-T infusion. Representative tumors from the indicated treatment groups are shown. Original magnification, ×20. Scale bars: 50 μm. **G** Mice that achieved complete regression (CR) against OSRC-2 for about 45 days and age-matched treatment-naive mice were subcutaneously inoculated with OSRC-2. Tumor growth for mice is shown (n = 6 for PBS and Ad5Ki67-C3 + CAR-T group, n = 3 for Ad5Ki67-C2 + CAR-T group). **H** Kaplan-Meier survival curve of rechallenge mice. Data are from the experiment shown in (**G**). **I** Body weights were measured. Data are shown as the mean ± SD. *P < 0.05; **P < 0.01, ***P < 0.001, ns means not significant. All scale bars = 50 μm.

Next, to examine whether localized activation of CAR-T cell by AdKi67-C3 affected systemic response, we conducted a bilateral OSRC-2 model in which NCG mice had subcutaneous tumors inoculated into both flanks (Fig. 5A). The results showed that compared with PBS, CAR-T or OAV mono treatment group could inhibit subcutaneous tumor growth at the initial stage, but the efficacy was limited. All tumors in mice that had been treated with AdKi67-C3 + CAR-T disappeared, and the contralateral tumors were also completely regressed. There were 3 of six mice achieved complete tumor regression (CR) in AdKi67-C2 + CAR-T treatment group. There was significant difference for mice tumor volume in AdKi67-C3 + CAR-T or AdKi67-C2 + CAR-T group compared with CAR-T mono treatment group (Fig. 5B). AdKi67-C3 + CAR-T or AdKi67-C2 + CAR-T had the longest time of survival compared to other groups (Fig. 5C). The CAR-T cells number in blood was detected by flow cytometry after CAR-T cells infusion on the indicated time. As shown in Fig. 5D, there were more CAR positive T cells in blood from AdKi67-C2 + CAR-T or AdKi67-C3 + CAR-T treated mice than CAR-T. The statistic assay was performed at 4-week time point after CAR-T cells infusion. It indicated that AdKi67-C3 promoted the proliferation and presence of CAR-T cells in vivo and evaluated the antitumor function of CAR-T cells. These data suggested that local injection of AdKi67-C3 has the potential to sensitize B7H3-CAR-T cells at both virus-injected and distant tumor sites.

Aiming to explore the detailed function of how OAVs affected tumor-infiltrating lymphocytes (TILs), 2 mice were sacrificed, and bilateral tumor tissues were collected on day 5 after CAR-T infusion. The gene expression was identified in injected or distant tumor mass. Elisa data showed that CCL5 was higher in both right and left tumor of AdKi67-C3 + CAR-T group and IFN-γ was higher in all AdKi67-DsRed, AdKi67-CCL5, AdKi67-C2 or AdKi67-C3 combined with CAR-T group. High level of IL-12 expression was detected in AdKi67-C2 or AdKi67-C3 combined with CAR-T group (Fig. 5E). Infiltration of CAR-T cells was determined by immunohistochemistry. As shown in Fig. 5F, there were more CD3^+^ cells in the right tumor mass than the left tumor mass for each combination group. The number of CD3^+^ cells within tumor of AdKi67-C3 + CAR-T group was the largest. It indicated that AdKi67-C3 efficiently promoted the infiltration of CAR-T cells in injected or untreated abscopal tumors.

The mice cured from AdKi67-C2 + CAR-T or AdKi67-C3 + CAR-T groups were also rechallenged with the same OSRC-2 tumor cells. The long-term persistence of antitumor memory existed, since the re-inoculated tumors were completely rejected within 5 weeks, but not in control group (Fig. 5G). The mice survived healthy all the time till the end of the experiment (Fig. 5H). No obvious therapy-associated side effects, indicated by body weight, were observed (Fig. 5I). These findings confirm that AdKi67-C3 enhanced CAR-T function to remove distant tumor lesions.

### The efficacy of local AdKi67-C3 injection combined with CAR-T cells in the treatment of distal intractable tumors

**Fig. 6:**
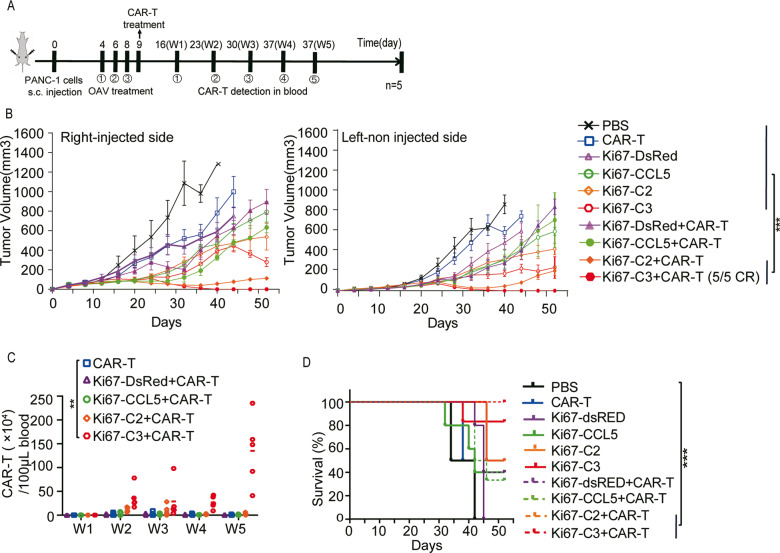
The efficacy of local OAV-C3 injection combined with B7H3-CAR-T cells in the treatment of distal intractable tumors in PANC-1 model. **A** Schematic presentation of the experimental setting. **B** Tumor volume in time. Data show means ± SD; n = 5. P values were calculated with two-way ANOVA. **C** B7H3-CAR-T cell number within the populations of CD3^+^ immune cells at different stages were detected by flow cytometry. Data show means ± SD; n = 5. **D** Animal survival curve. *P* values were calculated with log-rank test. *P < 0.05, **P < .0001.

Since AdKi67-C3 + CAR-T combination therapy had exciting outcome in two tumor models, we wanted to check its antitumor activity in a “cold” tumor model. The bilateral subcutaneous tumor model was constructed with pancreatic cancer cell line PANC-1, which had poor immunotherapy effect and high malignancy. The treatment strategy was shown in Fig. 6A. In the PANC-1 model, AdKi67-C2 + CAR-T or AdKi67-C3 + CAR-T group had better tumor inhibition ability compared with other groups. However, in AdKi67-C2 + CAR-T group, the injected right and distant left tumor volumes of mice decreased significantly in the early stage but increased at the later stage. Rather, the bilateral tumors of the OAV-C3 + CAR-T group eliminated in the early stage and were no recurrence (Fig. 6B). We observed significantly higher number of CAR-positive T cells in blood of AdKi67-C3 + CAR-T treated mice compared to other treatment groups. There was still a high value of CAR^+^T cells till 5 weeks after CAR-T cells infusion (Fig. 6C). Accordingly, AdKi67-C3 + CAR-T treatment significantly prolonged the survival period compared with PBS group (Fig. 6D). These data showed that AdKi67-C3 treatment enabled the activation of relevant immune-stimulating genes to increase the efficiency of B7H3-CAR-T and led an effective in vivo tumor regression.

## Discussion

CAR-T therapy in hematological malignancies has outstanding efficacy, triggering a profound impact on clinical cancer treatment. A number of studies have attempted CAR-T cell therapy as a treatment for solid tumors [33–35]. However, due to the complex molecular microenvironment and the lack of specific antigen targets in solid tumors, the application of CAR-T cell therapy in solid tumors faces tough challenges [36]. According to our results, low dose of B7H3-CAR-T cells had partial tumor inhibition in animal models but failed to induce a significant and persistent antitumor efficacy. The proliferation and survival might be not sufficient for tumor control. Recent years many therapies have been developed to improve the persistence and therapeutic efficacy of CAR-T cells against solid tumor [37, 38]. However, many inhibitory factors exist in the TME, more efforts may be required to achieve more expected therapeutic effects in clinical trials.

Oncolytic viruses (OVs) not only play a direct tumor lytic effect but also activates the host systemic immune system [39]. OVs could also remodel the immunosuppressive TME through several mechanisms and thus indirectly enhances the activation and function of immune cells in solid tumors [40]. It is worth noting that OVs can also be the effective vectors to delivery the immune modulators into tumors for further modification of TME [41]. OVs carrying genes encoding inflammatory cytokines are expected to enhance antitumor immune responses in TME, and a number of OVs have been evaluated for several cancers [42, 43]. In this manuscript, the 5-serotype adenovirus was used as the delivery vector to construct a parental oncolytic adenoviral vector AdKi67. Oncolytic adenoviruses AdKi67-DsRed (not arming with cytokine or chemokine), AdKi67-CCL5 (arming with a chemokine), AdKi67-C2 (arming with 2 cytokines), and AdKi67-C3 (arming with 3 genes) all intrigue the CAR-T proliferation while they used in combination therapy in NCG mice. This indeed supported that OV could modify the TME through certain mechanisms.

Inadequate CAR-T infiltration is associated with insufficient expression of T cell chemokines at the tumor site [15]. We did an analysis to explore the difference of T cells between in peripheral blood and infiltrated into tumor in patients. It showed that chemokine receptors expression such as CCR5 and CXCR3 was significantly higher in CD3^+^T cells infiltrated in tumors compared with that in blood. It suggested the key role of chemokines on recruiting immune cells in human cancers. Other studies also showed that transfection of tumor cells with CCL5 can induce T cells to infiltrate tumor [27, 44]. However, methylation of CCL5 mRNA in most tumor cells leads to decreased expression of CCL5. Therefore, CCL5 in the tumor microenvironment might be not sufficient to induce CAR-T invasion, requiring external supplementation. Our data showed that CCL5 carryed by AdKi67-C3 or AdKi67-CCL5 did induce more CAR-T cells infiltrating into tumor mass in tumor-burden mice.

Interleukin-12 (IL-12) is a potent, pro-inflammatory type 1 cytokine. IL-12 is responsible for inducing and enhancing cell-mediated immunity [28]. IL-12 has been shown to not only stimulate Th1 cell differentiation, enhance T and NK cell toxicity, but also inhibit or reprogram immunosuppressive cells, including tumor-associated macrophages (TAM) and medullary suppressor cells (MDSC) [45]. IL-12 expressed by dendritic cells can also increase the amount of IFN-γ expressed by CTL cells, and the increased IFN-γ can promote IL-12 expression, thus forming a positive feedback loop and enhancing antitumor activity [27, 46]. Our data showed that co-expression of IL-12 and IFN-γ mediated by oncolytic adenovirus AdKi67 increased the proportion of IFN-γ-positive CAR-T cells while co-culturing with tumor cells.

Combination therapy with CAR-T cells and oncolytic viruses is a new era in cancer immunotherapy. There are many different oncolytic viruses combing with multiple tumor-targeted CAR-T cells. The combination of HER2/neu CAR-T cells and oncolytic adenovirus-expressing anti-PD-L1 and IL-2 remarkably improved survival compared with monotherapy in head and neck squamous cell carcinoma xenograft mice [47]. Adenovirus AdC68-TMC-tCD19-mediated specific tumor tagging facilitates CAR-T therapy against antigen-mismatched solid tumors [48]. AdC68-TMC-tCD19 could mediate universal tag expression and functional immunological synapse formation between CAR-T and cancer cells. However, so far, few preclinical and clinical studies have been conducted on investigating the synergistic effects of CAR-T cell therapy and cytokine-armed OVs. In ClinicalTrials.gov, there is a Phase 1 study investigating the safety and efficacy of HER2 chimeric antigen receptor specific cytotoxic T lymphocytes (HER2 specific CAR T cells), in combination with intra-tumor injection of CAdVEC, an oncolytic adenovirus that is designed to help the immune system including HER2 specific CAR T cell react to the tumor (Clinical NCT number: NCT03740256).

In this study, we confirmed that oncolytic adenovirus AdKi67-DsRed, AdKi67-CCL5, AdKi67-C2, or AdKi67-C3 did not affect the proliferation of CAR-T cells in vitro. The supernants from AdKi67-CCL5, AdKi67-C2, or AdKi67-C3 infected tumor cells had enhanced the function of CAR-T cells. The synergistic antitumor effect of AdKi67-C3 combined with CAR-T was further observed in vitro. Mono CAR-T cell therapy or oncolytic adenovirus therapy had less therapeutic benefit. The in vivo data showed that oncolytic adenovirus AdKi67-DsRed, AdKi67-CCL5, AdKi67-C2, AdKi67-C3 or B7H3-CAR-T cell monotherapy had partially inhibited tumor growth. However, AdKi67-DsRed, AdKi67-CCL5, AdKi67-C2, or AdKi67-C3 combined with B7H3-CAR-T exhibited a significantly improved antitumor efficacy, especially for AdKi67-C3 + CAR-T treatment, with long-term tumor-specific immune surveillance in solid tumors. AdKi67-C3 had increased the proliferation and survival of CAR-T in vivo and persisted for a long time. There seemed to be a higher proportion of memory CAR-T cells since the mice inoculated with the same tumor were complete tumor regression. CAR-T cells in other combination group were gradually disappeared after treatment. AdKi67-C3 further enhanced the antitumor efficiency of B7H3-CAR-T when the bilateral tumor burden was suppressed, and the cure rate was robustly 100%. Our results underscored the point that AdKi67-C3 could be used as an adjuvant for CAR-T cell therapy. However, the limitation of our study was that these studies were done using human tumors with human T cells in NCG mice. As we know the interaction of the oncolytic adenovirus with the host TME is critical, but these mice didn’t have a normal immune system that would be activated by the oncolytic adenovirus. The real-world activities of immune response can’t be assessed in the used mice model.

In conclusion, we found that the constructed recombinant bioenhancer of AdKi67-C3 could significantly improve the function of B7H3-CAR-T cells to completely clear local and distant tumors and induce long-term protection from preventing tumor recurrence in mice. This finding may provide a new option for clinical treatment.

## Materials and methods

### Cell lines and viruses

The human cancer cell lines Du145 (prostate cancer), OSRC-2 (renal cancer), and PANC-1 (pancreatic cancer) were obtained from the American Type Culture Collection (Manassas, VA) and the RIKEN (Tokyo, Japan) respectively. Du145, OSRC-2, and PANC-1 cells were maintained in Dulbecco’s modified Eagle’s medium. All media were supplemented with 10% fetal bovine serum, 100 U/mL penicillin, and 100 mg/mL streptomycin. Cells were maintained at 37°C in a humidified atmosphere with 5% CO_2_.

Engineered oncolytic adenovirus was constructed previously in the backbone of a tumor-selective oncolytic adenovirus ZD55 vector, in which E1A-55KD had been deleted, in addition, tumor-specific promoter Ki67 was used to drive the adenoviral E1A expression. Interested gene DsRed, human or mouse CCL5, IFN-γ, or IL-12 expression cassette was inserted into the E3 region of adenovirus as designed respectively. HEK293 cells were used to amplify virus OAV, and the titers were measured by tissue culture infection dose 50 (TCID_50_) methods.

### Primary T cell isolation, culture, and transduction

Peripheral blood of healthy donors was obtained from Xuzhou Medical University. Informed consent was obtained from all human subjects included in this study. Peripheral blood mononuclear cells from healthy donors were collected and activated by human T-activator CD3/CD28 Dynabeads (Gibco, Life Technologies) following the manufacturer’s protocol. T cells were cultured in X-vivo 15 (LONZA, Walkersville, MD) supplemented with 1% human serum albumin, 1% P/S, 10 ng/ml human IL-7 and 5 ng/ml human IL-15 (PrimeGene, China). The medium was replaced every other day. Forty-eight hours post activation, T cells were transduced with B7H3-specific CAR retroviruses with spin infection (30°C, 1500 g, 1.5 h). Three days after infection, T cells were analyzed for CAR-positive population percentage by flow cytometry.

### Elisa

Cells (3×10^5^ in six-well plate) were infected with different OAVs at MOI = 20 for 24 h. The culture medium was removed, and the condition culture medium was collected on 24 h or 48 h. The CLL5, IFN-γ or IL-12 protein expression was determined by Elisa according to the manufacturer’s protocol. The tumor tissue lysates form different treatment groups were prepared, and the protein expression was determined by Elisa according to the manufacturer’s protocol.

### Virus replication assay

Logarithmically growing cancer cells (10^5^ cells per well) were cultured in six-well plates for 24 h. Later, cells were infected with different OAVs at MOI = 20 respectively for another 2, 24 or 48 h. Then, cells were lysed through three cycles of freeze and thaw. The titers of viral progenies were quantified on HEK-293 cells with the TCID_50_ method. The replication multiples of adenovirus in the tested cancer cells were normalized with that at the beginning of infection (2 h post infection).

### T cell trafficking assay

CAR-T cell trafficking assays were performed as previously described using 5 μm pore 24-well transwell plates (Corning Life Science). CCL2 (100 ng/mL), CCL3 (100 ng/mL), CCL4 (10 ng/mL), CCL5 (100 ng/mL), CCL8 (5 ng/mL), CCL14 (100 ng/mL), CCL19 (50 ng/mL), CXCL8 (100 ng/mL), CXCL10 (20 ng/mL), CX3CL1 (50 ng/mL), CCL3L1 (100 ng/mL), CXCL16 (200 ng/mL) or the condition medium from tumor or infected tumor cells was added into the lower chamber. The percentage of migrating cells was calculated as follows: 100×[cell count of experimental sample – cell count of negative control] / [cell count of positive control – cell count of negative control].

### xCELLigence

The xCELLigence real-time cell analyzer (RTCA) system instrument (ACEA Biosciences) was used for impedance experiments to determine tumor cell killing according to the manufacturer’s protocol. Briefly, tumor cells were plated at 10,000 cells per well, 12 h later, following by adding MOIs of OAV or CAR-T cells with an effector: target ratio of 1:2. For CAR-T cells–mediated tumor killing or combination with OAV assay, CAR-T cells and the indicated MOI of OAV were added to the cells at the same time. The combination index was calculated according to a modified Burgi’s formula. The formula: Q = E_A+B_/ (E_A_ + E_B_ –E_A_·E_B_) (Note: E_A_ is the effect of drug A, E_B_ is the effect of drug B, and E_A+B_ is the effect of combination drug).

### Flow cytometric analysis

To analyze the expression of DsRed in different cell lines infected with AdKi67-DsRed, cells were collected on 1- or 2-days post infection to FACS analysis. Human CAR-T cells obtained from the CAR-T-infused NCG mice PBMC or tumors were stained with the following antibodies: human CD3-APC (Biolegend), Human Recombinant B7-H3 Protein (ECD, hFc Tag, Sino Biological), Goat anti-Human IgG Fc Secondary Antibody, PE (Thermo Fisher Scientific). For IFN-γ staining, cells were fixed and permeabilized using the BD Cytofix/Cytoperm Fixation/Permeabilization Solution Kit according to the manufacturer’s protocol (BD Biosciences). Cells Samples were also analyzed on a BD FACS Canto II flow cytometer (Becton Dickinson). Data were analyzed by FlowJo version 10 software (Tree Star Inc.).

### CFSE cell labeling

To detect the effect of oncolytic adenovirus OAV on CAR-T cell proliferation, B7H3-CAR-T cells were labeled with CFSE (carboxy fluorescein diacetate, succinimidyl ester) at a final concentration of 10 mM and then different MOIs of OAV was added respectively. After 72 h, B7H3-CAR-T cells were collected, and the proliferation of B7H3-CAR-T cells was detected by flow cytometry.

### In vivo tumor studies

All animal experiments were performed under protocols approved by Xuzhou Medical University Institutional Animal Care and Use Committee. For human tumor xenograft studies, DU145/OSRC-2/PANC-1 cells (2×10^6^cells per mouse) were prepared and injected subcutaneously into the left and/or right flank of male NCG mice (purchased from Nanjing University Model Animal Institute, China). Tumor growth and mice body weight were monitored two to three times per week by caliper measurement. Tumor volume was calculated by the formula length×width^2^/0.5. Once tumor volumes reached about 50 to 100 mm^3^, PBS, OAV was intratumorally administered every other day for a total of three injections at 10^9^ PFU per mouse. For combination therapy or CAR-T monotherapy, B7H3-CAR-T cells (2×10^6^ cells per mouse) were injected via tail vein on the next day after the last OAV treatment. For B7H3-CAR-T cell detection in blood, bleeding was performed on day 7, 14, 21, 28, 35 after CAR-T infusion from mouse for each group. For measurement of intratumoral cytokines, tumor samples were collected and immediately frozen in liquid nitrogen. Intratumoral IL-12, IFN-γ, CCL5 was quantified using Elisa Kit (Dakewe Biotech, China) respectively.

In rechallenge studies, mice previously cured of combination therapy or age-matched treatment-naive mice were inoculated with same tumor cells subcutaneously. Tumor volumes were monitored twice a week by caliper measurement.

### Immunohistochemistry staining analysis

The tumor tissue was fixed in 10% formalin, embedded in paraffin and cut into 3 mm sections. Deparaffinized tumor sections were treated with primary antibody for CD3. After incubation with a secondary antibody, tissue sections were then counterstained with hematoxylin. Representative sections were stained with Masson trichrome and then observed by light microscopy.

### Expression profiles of T cells in blood or renal cancer clinical specimen

The matched T cells in peripheral blood or CD3-positive tumor-infiltrating lymphocytes were purified form 3 renal cancer patients. All the samples were collected and studied under a protocol approved by the Affiliated Hospital of Xuzhou Medical University Review Board. Informed consent was obtained from all human subjects included in this study. RNA was extracted. Isolated RNA was quantified to analyze the gene expression (Beijing CapitalBio Technology Co. Ltd.).

### Statistical analysis

Statistical analysis was conducted with GraphPad Prism 8. Data are presented as means ± SEM. Statistical comparisons between groups were performed using the one-way ANOVA test to calculate *p* value. The survival curve was obtained by Kaplan-Meier plot, and a two-sided log rank test was applied for mouse survival test; *p* < 0.05 was considered statistically significant for all analyses. (*p < 0.05; **p < 0.01; ***p < 0.001).

## Data Availability

The datasets generated during and/or analyzed during the current study are available from the corresponding author on reasonable request.
